# Multi-slotted airfoil design for enhanced aerodynamic performance and economic efficiency

**DOI:** 10.1038/s41598-025-87000-z

**Published:** 2025-02-04

**Authors:** Mohamed A. Aziz, Mohamed A. Khalifa, M. A. Abdelrahman, Haitham Elshimy, Ahmed M. Elsayed

**Affiliations:** 1 Mechanical Engineering Department, Faculty of Engineering, Suez University, P.O. Box 43221, Suez, Egypt; 2 Mechanical Engineering Department, Institute of Aviation Engineering and Technology, Giza, Egypt; 3 Mechanical Engineering Department, Faculty of Engineering, Shoubra Faculty of Engineering, Benha University, Benha, 11629 Egypt; 4 Space Navigation Department, Faculty of Navigation Science and Space Technology, Beni Suef University, Beni Suef, Egypt; 5 Mechanical Engineering Department, Faculty of Engineering, Fayoum University, Fayoum, 63514 Egypt

**Keywords:** Passive flow control, Multi slotted airfoil, Wind turbine, NACA 23012C, Numerical study, Renewable energy, Aerospace engineering, Mechanical engineering

## Abstract

Recently, slotted airfoils have been introduced as a passive flow control approach. The slotted airfoil method resulted in stall delay and enhanced the lift coefficient. The single-slot airfoil is unable to delay stall if the flow is injected downstream of the separation point at the stall angle of attack. A multi-slot airfoil ensures air is injected along the airfoil suction side, delaying stalls over a large range of AOA. The current study focuses on enhancing wind turbine blades’ efficiency by utilizing a novel multi-slot NACA23012C airfoil design as a passive control approach. A numerical study of the optimal grid number was carried out, followed by validating the numerical model with previous experimental results in the literature. The numerical study is followed by a study of the effect of the number of airfoil slots: one, two, three, four, five, and six. The characteristics of the flow field were analyzed to explain the benefit of applying multi-slotted on the aerodynamic performance of an airfoil with a high AOA at Reynolds number 2.74 × 10^5^. The findings showed a significant improvement in the lift coefficient values and the delayed stall AOA for multi-slot airfoils compared to the clean and single-slot airfoils. Increasing the slots number is effective up to four slots. The four-slot airfoil improved lift by 15.8%, and the two slots achieved a 22.31% CL/CD increase. Future work could optimize slot geometry, validate findings experimentally, and study dynamic and 3D effects.

## Introduction

Wind turbines are used to convert kinetic energy into electrical energy through horizontal or vertical axis blades. The blade section of the wind turbine is shaped like an airfoil. The performance of the wind turbine depends on the aerodynamic performance of the airfoil under different operating conditions. Therefore, improving airfoil performance is of utmost importance, especially under specific operating conditions in which airfoils are subjected to high AOAs.

Numerous flow control methods, apart from airfoil control, have lately been successfully applied to wind turbines. Passive flow control is one of the successful methods that proved successful in improving the aerodynamic performance of the airfoil in many research studies. Aboelezz et al.^1^ have performed numerical and experimental investigations for the design of the airfoil with the guided vane. They indicated that the guided vane could be fitted with exciting wind turbines to enhance its performance. Their results showed an increase in measured output power of up to 26% due to introducing a guided vane airfoil to the wind turbine. Roy et al.^2^ have performed experimental and computational investigations for the NACA 4415 airfoil with the addition of protrusions in the leading edge at a Reynolds number (Re) of 1.5 × 10^5^. They considered spherical and triangular shapes as the leading-edge protrusions. The addition of protrusions was able to delay the flow separation. At higher AOAs (in the post-stall region), spherical protrusions enhanced the lift generation of the airfoil blade. Chinnappa et al.^3^ have compared the effect of Gurney flaps on different flow characteristics. They studied the effect of design geometric parameters of the Gurney flap, specifically flap length and flap position, on the airfoil NACA 23112. They focused on finding the optimum design parameters. Their results indicated that introducing Gurney flaps enhanced the airfoil performance at higher AOAs. Julian et al.^4^ have analyzed numerically the effect of slat size as a passive flow control at a Reynolds number of Re = 10^6^. The slat geometry is used as the NACA 6641 airfoil. Based on their results, the slat size has a considerable effect on the aerodynamic performance of the airfoil. They concluded that slat could increase the lift coefficient as well as delay a stall to a higher AOA. On the other hand, slat size also influences increasing the drag coefficient. Genc et al.^5^ have introduced a pre-stall control mechanism by using roughness material over the airfoil surface. They showed that the roughness of the material over the airfoil surface may lead to an increase in flow momentum. As well as causing a bypass transition to occur. It also postponed the stall and enhanced aerodynamic performance.

Evidence of passive slots in the airfoil for increasing their lift is well known. The increase in lift is accompanied by a consistent increase in drag so that the overall aerodynamic efficiency is, at most, slightly enhanced. Acarer^6^ has numerically studied the leading-edge slot type applied to the DU12W262 airfoil. The effect of leading-edge slots was verified on vertical and horizontal axis wind turbines (VAWTs & HAWTs). The results indicated a 16% increment in lift-to-drag ratio compared with the standard airfoil. The increment in power coefficient for HAWT was 7.5% and 9.6% for VAWTs. Additionally, passive flow control methods have been explored, such as adapting the airfoil’s shape to an unusual one using vortex generators, cavities in the airfoil surface, winglets, wavy-shaped airfoils, and the application of stepped configurations at the top or bottom of the wing^7^. Belamadi et al.^8^ numerically explored the enhancement of wind turbine airfoil aerodynamic characteristics at stall conditions by applying a passive synthetic jet. Their results indicated that the jet enhanced the aerodynamic forces in a certain range of AOA. Bhavsar et al.^9^ have introduced a numerical study on applying a slot as a control device to the DU-99-W-405 airfoil and studied its effect on the lift and drag coefficients. They concluded that the optimum position for the slot corresponds to improvements of 68.8%, 36.9%, and 116% in C_L_, C_D_, and C_L_/C_D_ values, respectively. Aziz et al.^10^ have performed a numerical investigation of airfoil flow control using passive air jets. They studied the generation of a passive jet from the pressure side to the suction side to improve the airfoil characteristics. They concluded that the maximum lift is attained with the jet flow being normal to the suction side surface, but this comes with the drawback of the high drag. The maximum lift-to-drag ratio was associated with the synthetic jet being positioned at a 43% chord and a 30° jet angle.

Moshfeghi et al.^11^ have numerically investigated the effects of a passive flow control method on the aerodynamic performance of a HAWT by splitting its blades along the span. Their results revealed that torque values are delicately dependent upon split position and flow injection angle for an attached flow case. On the other hand, for a moderately separated flow case, results show that the split location may impose either positive or negative effects on torque generation. Beyhaghi et al.^12^ have introduced a study on the effect of a thin span-wise rectangular conduit drilled near the leading edge of a finite-span cambered airfoil on the overall aerodynamic performance. They used NACA 4412 as the baseline airfoil profile. They studied the effect of the slot’s width, inlet angle, and vertical position. Their results included a lift coefficient enhancement of up to 30% while the drag slightly increased. Ni et al.^13^ have studied the effect of internal slots on a NACA 634-021 airfoil blade to allow flow through the slot from the pressure side to the suction side of the blade on flow separation and stall delay. They noticed an increase in lift and a decrease in drag at significantly high AOAs. Their results indicated an increase in maximum lift coefficient equal to 58% and an increase in maximum lift-to-drag ratio equal to 14% could be attained with the optimum design case. Mohamed et al.^14^ have investigated the effect of employing slotted airfoils as turbine blades on performance and starting characteristics. They considered the slot parameters—slot location, angle of inclination, and dimensions—while optimizing the modified NACA 0018 airfoil with the slot. Their results showed that slotted airfoil wind turbines have a lower tip speed ratio compared to baseline turbines. Also, the airfoil slot delays the separation at high AOAs. Coder et al.^15^ have examined the slotted S207 airfoil using requirements derived from a transonic, truss-braced wing commercial aircraft configuration. Their investigations showed strong potential for meeting mid- and far-term goals for reducing aircraft fuel and energy consumption.

Recently, Akhter et al.^16^ used CFD to analyze an S809 airfoil with a mid-chord inclined curved slot, showing enhanced performance at α > 6°, with up to 1.3 × lift, 0.5 × drag, and 3.7 × glide ratio improvements. Slot-induced flow control reduced separation, turbulence, and noise by 14 dB at α = 17°, demonstrating its effectiveness for wind turbines. Jaffar et al.^17^ numerically investigated passive air-blowing effects on the DU97-W-300 airfoil’s near-wake region, focusing on slot geometry (height, width, inlet position). The optimized design (4 mm width, 0.025%c inlet) reduced drag and turbulence kinetic energy, enhancing aerodynamic performance and potentially reducing aerodynamic noise at high AOA (α > 16). Aziz et al.^18^ experimentally investigated pulsed suction air jets for flow control on a NACA 0012 airfoil at Re = 0.5 × 10^5^–1.4 × 10^5^. Results showed lift coefficient improvements (up to 16.33% at 9 Hz) and delayed stall by 1° at Re = 1.1 × 10^5^, while drag reduction reached 27.4% at 9 Hz and Re = 1.4 × 10^5^.

Table 1 highlights the key characteristics and most important results from other recent studies on slotted airfoils for passive aerodynamic control of small wind turbines based on the literature, emphasizing the gap and novelty of the current research. Based on the presented literature, all researchers are interested in studying the one-slotted airfoil geometry. Its effect fails if the flow is injected downstream of the separation point. The location of the separation point changed with AOA and the wind speed. The current study focused on multi-slotted passive flow control to cover the overall suction side surface with energized flow from the pressure side near the stagnation point. The proposed multi-slotted airfoil has a positive economic impact as well because emptying many openings and air flow paths inside the airfoil reduces the airfoil area and thus the amount of raw materials used in manufacturing the turbine blades, which has a positive impact on lowering the cost of wind turbine manufacturing.

**Table 1:** Summary of related previous studies in the literature compared to current work.

Ref	Type of study	Airfoil	Reynolds number	AOA (°)	Slot shape parameters	No. of slots	Enhancement
Acarer^6^	(CFD) simulations	DU12W262	Not mentioned	− 20° to 20°	5 parameters	One	16% peak C_L_/C_D_ improvement overall α-C_L_/C_D_ rise
Belamadi^8^	Numerical study	S809 airfoil	1.0 × 10^6^	10° to 20°	3 parameters	One	The improvement of flow control obtained with high AOA
Bhavsar^9^	Numerical study	DU-99-W-405	8.22 × 10^5^	− 8° to 32°	2 parameters	One	The optimum C_L_, C_D_, and C_L_/C_D_ reached 68.8%, 36.9%, and 116% respectively
Aziz et al.^10^	Numerical study	NACA 23012C	1.0 × 10^6^	0° to 26°	Jet angle, jet location & slot width	One	An increase in the C_L_/C_D_ by 32.0% Delays the lift coefficient stall by Δα = 7°
Moshfeghi et al.^11^	Numerical study	S809 airfoils	From 3.0 × 10^5^ to 1.0 × 10^6^	0° to 20°	Split widths	One straight slot	Max. torque enhancement 10.7%
Beyhaghi^12^	Numerical study and experimental case study	NACA 4412	1.6 × 10^6^	0° to 16°	Slot’s width, inlet angle & vertical position	One	Power generation improvement of 8–12%
Ni et al.^13^	Numerical study and experimental case study	NACA 634-021	1.0 × 10^5^	0° to 30°	Slot width & inclined angle	One	58% increase in maximum lift coefficient 14% increase in maximum lift-to-drag ratio
Mohamed et al.^14^	Numerical study	NACA 0018	2.5 × 10^5^	0° to 30°	Slot location, angle of inclination, & dimensions	One	Generate higher torque & t delays the separation
Coder et al.^15^	Theoretical predictions	S207 & CRM.65	13.2 × 10^6^	− 4:16	Fixed slot	One	Enhanced the aerodynamic performance, reducing aircraft fuel/energy consumption
Current study	Numerical study	NACA 23012C	2.74 × 10^5^	0 :28	Slot Location & Slot numbers	One, Two, Three, Four, Five & Six	Optimum number of slots is achieved. Delay stall by 12°
							Increase C_Lmax_ (15.8%)
							Reduction in minimum drag (22.1%)
							Increase C_L_/C_D_ (22.31%)

The paper is structured according to the following points:

- The definition of the new synthetic multi-slotted airfoil proposed in the current study represents the novelty of the work.
- Computational modeling includes grid-independent study and model validation with previous experimental work in literature.
- Numerical results and discussion in terms of global aerodynamics characteristics such C_L_, C_D,_ and C_L_/C_D_.
- Numerical results and discussion in terms of local aerodynamics characteristics such as pressure and velocity contours with streamlines, pressure coefficient distribution on the surface of the airfoil, and velocity profile along the suction side of the airfoil.
- Finally, the economic impact of using multi-slots inside the airfoils to reduce the size of the wind turbine blades and the cost of manufacturing the turbine was assessed.

## New synthetic slots definition

**Fig. 1:**
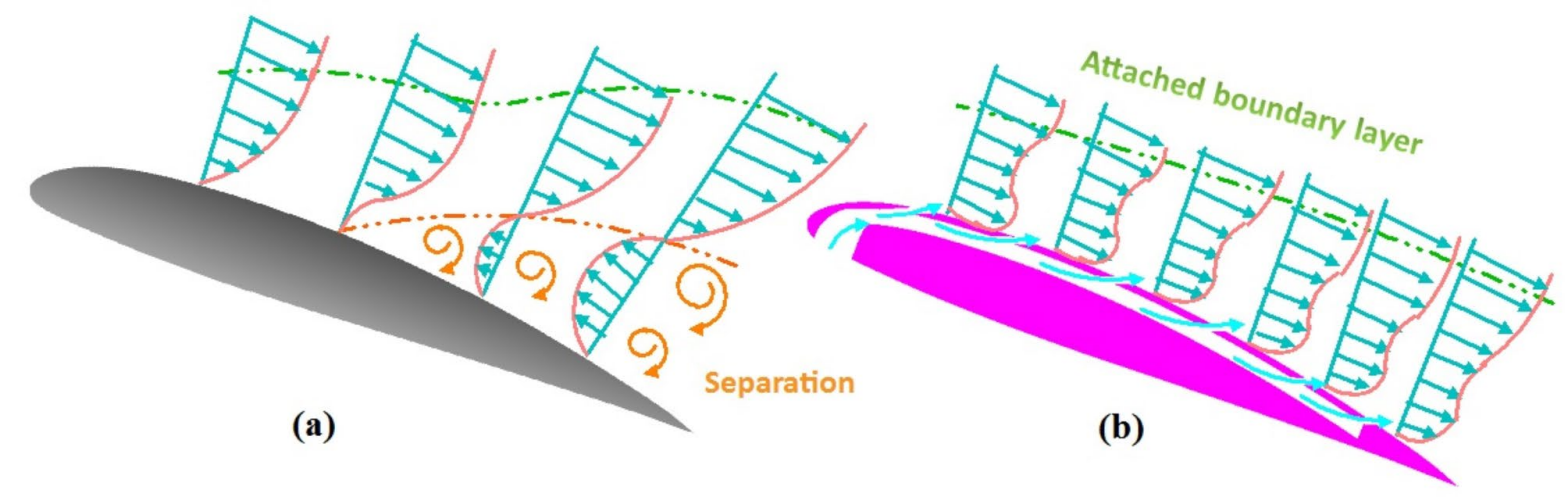
Airflow at high AOA for (**a**) clean airfoil with separated boundary layer and (**b**) multi-slotted airfoil with attached boundary layer.

The boundary layer is one of the most important aerodynamic characteristics of the airfoil, as it affects the resultant forces resulting from the difference in pressures between the two sides of the airfoil. The boundary layer is completely adjacent to the surface of the airfoil, starting from the leading edge, until the energy decreases because of the high adverse pressure gradient. A sudden increase in pressure gradient occurs at high angles of attack, resulting in the separation of the boundary layer adhering to the airfoil surface. This is followed by a deterioration in the lifting force and an increase in the drag force. The separation points change with increasing wind speed, angle of attack, or both. The separation points advance towards the airfoil’s leading edge as the angle of attack increases. Addressing this requires a robust system that covers areas where separation is likely to occur along the suction surface of the airfoil. The new design of the slotted NACA 23012C airfoil compared to the clean airfoil is presented in Fig. 1. In the absence of slots, separation occurs (Fig. 1a), while in the presence of slots, the air covers the entire surface of the airfoil (Fig. 1b), eliminating separation and reverse flow while supplying the air with energy drawn from the stagnation point or close to it.

**Fig. 2:**
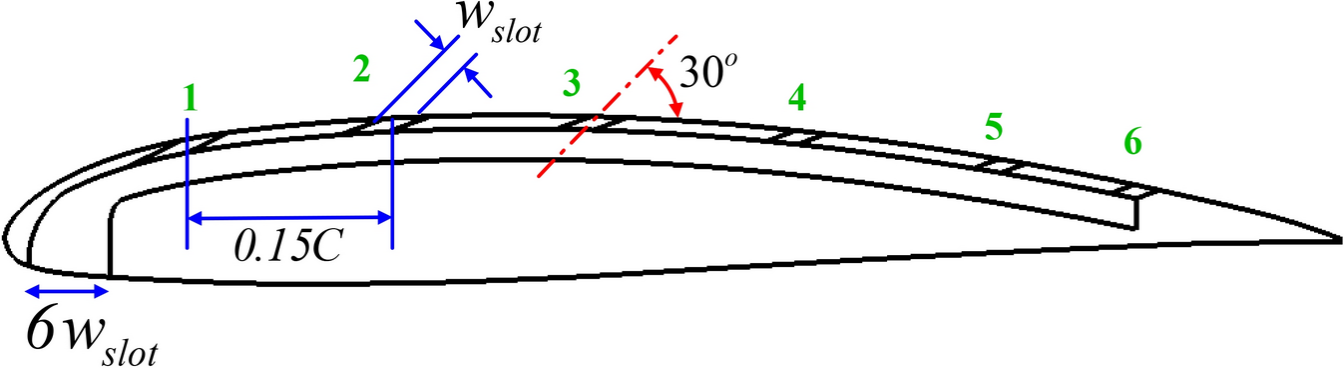
Airfoil with synthetic slots geometry definition.

Figure 2 shows the dimensions proposed in the current study, which govern the shape, location, and angle of each slot. The size of the slot, the angle of the slot, and the streamwise distance between slots are kept fixed throughout the study; they are based on a previous study^10^. In the case of a single slot airfoil, the first slot was started at a distance of 0.15C (where C is the chord length) from the leading edge, and the slots were increased in the direction of the trailing edge while maintaining a constant distance between each of the two slots equal to 0.15C to ensure that the upper surface of the airfoil was completely covered to the greatest extent possible. This was also found to somewhat prevent interference between each jet and the other so as not to weaken the effect of each jet. The current study deals with low speeds, less than 0.3 Mach number. Therefore, assuming the flow is incompressible, the slot inlet area was kept equal to the jet area to maintain the speed at the jet location. The jet angle was also maintained at 30° for all slots, which ensures flow adjacent to the airfoil surface for the flow coming from each slot. Table 2 summarizes slotted airfoil geometrical and flow parameters.

**Table 2:** Slotted airfoil geometrical and flow parameters.

Number of slots	One slot (1)	Two slots (1,2)	Three slots (1,2,3)	Four slots (1,2,3,4)	Five slots (1,2,3,4,5)	Six slots (1,2,3,4,5,6)
Slot width inlet	18 mm	18 mm	18 mm	18 mm	18 mm	18 mm
Slot width exit	3 mm	3 mm	3 mm	3 mm	3 mm	3 mm
Slot angle	30°	30°	30°	30°	30°	30°
Chord length	200 mm	200 mm	200 mm	200 mm	200 mm	200 mm
Angle of attack	16°:28°	16°:28°	16°:28°	16°:28°	16°:28°	16°:28°

**Fig. 3:**
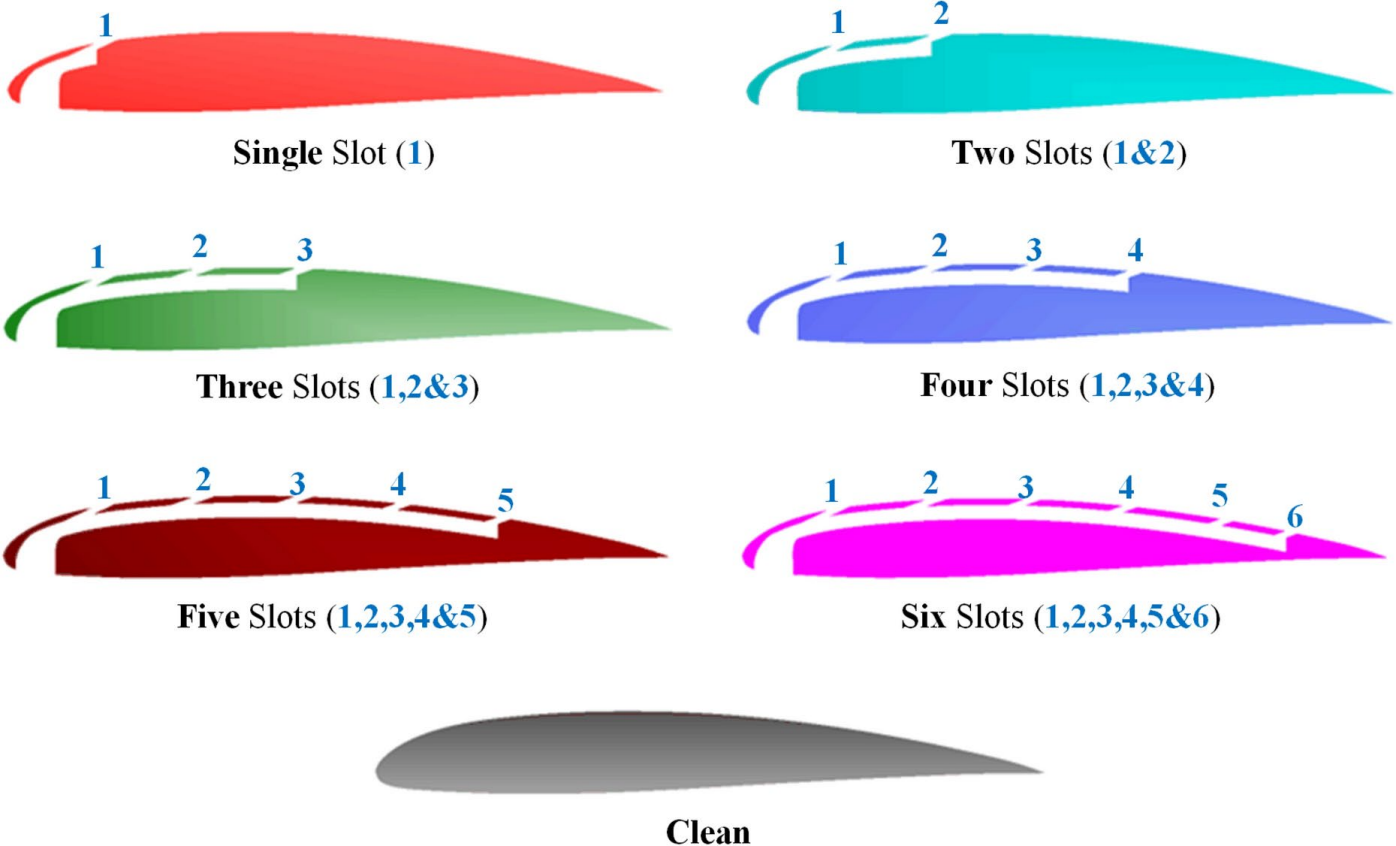
Various slotted airfoil configurations studied.

Figure 3 displays the shapes proposed in the current study for distributing slots while changing the number of slots and the clean airfoil. The withdrawn flow in the different shapes is from the pressure side near the stagnation point, where there is high pressure. The width of the withdrawn slot is fixed in the different cases at six times the width of each injected slot to compare the effect of changing the number of inject slots on the suction side.

## Computational modeling

Numerical simulations were performed using a commercial CFD solver (CFDRC). The geometry and grid creation were generated in the CFD-GEOM, while the CFD simulation was conducted in the CFD-ACE. The results are presented in the CFD-VIEW. All these modules are manipulated using the Python programming language. The solver is a pressure-based type that solves the compressible Navier–Stokes equations with SIMPLE (Semi-Implicit Method for Pressure Linked Equations). Moderate CFL was used in steady-state simulations for faster convergence. The solver allows the use of structured or unstructured grids for turbulent flows. Turbulence is modeled using RANS equations. The spatial accuracy is second-order when using a second-order upwind scheme. The convergence criteria for the simulations were 10E-5. The under-relaxation factor was 0.5 for all variables. The turbulence intensity equals 5%, and the turbulence length scale is 0.07 times L. The mass flux residuals for the simulation were 1E-4 kg/s. A finite volume method is used to discretize the governing equations in their vector form, Sect. 1.12 of^19,20^, and^21^.

Turbulence is characterized by chaotic, time-dependent fluctuations in velocity and other flow properties. Solving the full Navier–Stokes equations with all turbulence scales (Direct Numerical Simulation, DNS) is computationally expensive, so turbulence models are introduced to approximate the effects of turbulence. Commonly used models include RANS (Reynolds-Averaged Navier–Stokes). In the RANS framework, any flow variable, $\emptyset$, (such as velocity, V, or pressure, P), is split into a mean value (time-averaged), $\overline{\emptyset}$, and a fluctuating component (turbulent fluctuations), $\acute{\emptyset}$:

1 $$\emptyset = \overline{\emptyset} +\acute{\emptyset}$$

The goal of the RANS model is to solve for the mean flow quantities and introduce additional terms (Reynolds stresses) that account for the effects of turbulence.

The RANS incompressible continuity equation is:

2 $$\nabla \cdot \overline{V} = 0$$

The momentum equation with RANS decomposition introduces additional terms known as Reynolds stresses. The decomposed momentum equation becomes:

3 $$\rho \frac{{D\overline{V}}}{Dt} = - \nabla \overline{P} + \nabla \cdot \left( {\mu \nabla \overline{V}} \right) - \nabla \cdot \overline{\rho \acute{V}\acute{V}} + F$$

where $\overline{V}$ is the mean velocity vector, $\overline{P}$ is the mean pressure, and $\overline{\rho \acute{V}\acute{V}}$ is the Reynolds stress tensor which accounts for the turbulent fluctuations.

The Reynolds stress tensor $\overline{{{\uprho }\acute{v}_{i} \acute{v}_{j}}}$ introduces six new unknowns, representing the additional stress on the flow caused by the turbulent velocity fluctuations. These stresses are modeled through turbulence models (like the k-ε or k-ω models), rather than being directly solved. The Reynolds stress tensor is modeled using the Boussinesq approximation, which introduces the concept of turbulent eddy viscosity μ_t_ to relate the Reynolds stresses to the mean velocity gradients:

4 $$\overline{{{\uprho }\acute{v}_{i} \acute{v}_{j}}} = \mu_{t} \left( {\frac{{\partial \overline{{V_{i} }} }}{{\partial x_{j} }} + \frac{{\partial \overline{{V_{j} }} }}{{\partial x_{i} }}} \right) - \frac{2}{3}{\uprho }k\delta_{ij}$$

where μ_t_ is the turbulent viscosity and is computed using a turbulence model like k–ε or k–ω, k = 0.5 $\overline{{\acute{v}_{i} \acute{v}_{j}}}$ is the turbulent kinetic energy, *δ*_*ij*_ is the Kronecker delta (used for isotropic turbulence).

In the RANS energy equation, the fluctuating components contribute additional terms for turbulent heat flux. The time-averaged energy equation becomes:

5 $$\frac{{\partial {\overline{\text{Q}}}}}{{\partial {\text{t}}}} + {\Phi } + \nabla \left( {{\text{k}}\nabla {\overline{\text{T}}}} \right) - \nabla {\overline{\text{q}}}_{{\text{r}}} = {\uprho}\frac{{{\text{D}}\overline{{\text{e}}}}}{{{\text{Dt}}}} + {\overline{\text{P}}}\nabla {\overline{\text{V}}} - \nabla {\overline{\text{q}}}_{{\text{t}}}$$

where $\overline{q}_{t} = \overline{\rho c_{p} \acute{V}\acute{T}}$ is the turbulent heat flux, $\overline{T}$ is the mean temperature, *c*_*p*_ is the specific heat at constant pressure.

Using the eddy-diffusivity approximation, the turbulent heat flux is modeled as:

6 $$\overline{q}_{t} = \frac{{\mu_{t} }}{{P_{{r_{t} }} }}\nabla \overline{T}$$

where $P_{{r_{t} }}$ is the turbulent Prandtl number.

Table 3 presents the discretization schemes and methods chosen for key variables in the current simulation.

**Table 3:** Discretization schemes and methods for key simulation variables.

Variable	Discretization	Clarification of the selection
Momentum	Upwind scheme	This preferred for robustness and stability, especially in turbulent and complex flow fields
Pressure	Simple	This preferred choice for steady-state problems, providing a balance between robustness and computational efficiency
Turbulence	Second-order upwind	A more accurate representation of turbulence behavior is needed, especially in critical regions like boundary layers, near-wall regions, or separation zones

### Grid independent study

**Fig. 4:**
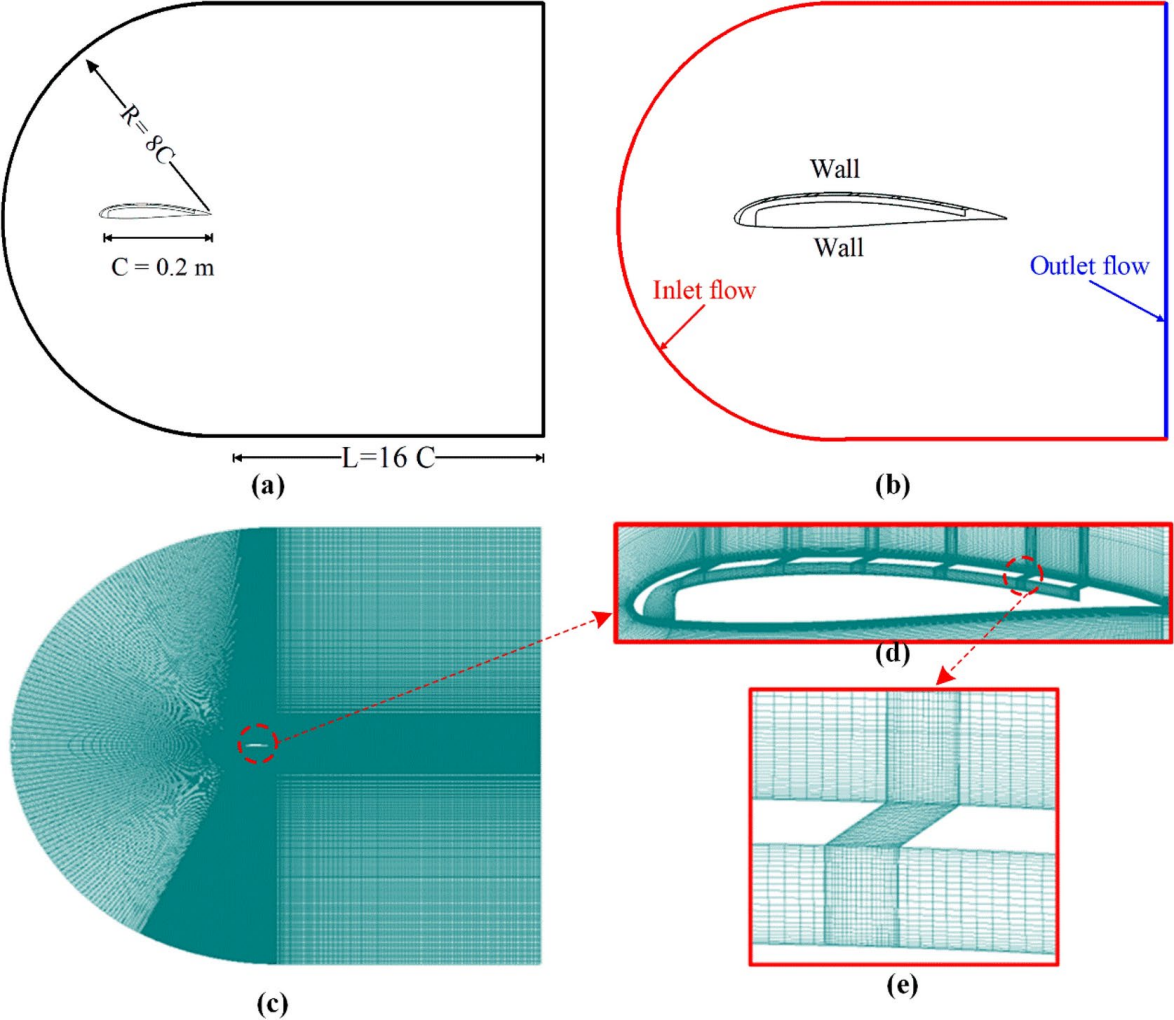
Schematic of (**a**) 2D airfoil CFD control volume, (**b**) CFD domain with boundary conditions, (**c**) structured grid for CFD entire domain, (**d**) structured grid around a slotted airfoil, and (**e**) structured grid through the slot.

Figure 4a shows the computational domain dimensions. The domain size was selected based on previous studies^22,23^. The distance downstream of the airfoil was kept at 16C, while the radius of the circular border at the entrance was 8C. The downstream length behind the airfoil has been doubled to include the rear vortices due to airflow separation at high AOA. Figure 3b displays the specified boundary conditions at the computation borders and the airfoil surface. The inlet flow is stated by sea level conditions Pa, Ta, and free stream velocity, V, equal to 20 m/s (Re = 2.74 × 10^5^) with different AOA ranging from 0° to 28°. The outlet flow is defined by a fixed pressure under sea-level conditions. Figure 4c–e present the computational grid proposed in the study while zooming in on the airfoil and near-slot zones. The inflation layer near the walls was offered to ensure the capture of the boundary layer. The smallest volume is 1.2E−09 m^3^, and the corresponding y + value is less than 1.

Figure 4b displays the specified boundary conditions at the computation borders and the airfoil surface. The inlet flow is stated by sea level conditions Pa, Ta, and free stream velocity, V, equal to 20 m/s (Re = 2.74 × 10^5^) with different AOA ranging from 0° to 28°. The outlet flow is defined by a fixed pressure under sea-level conditions. Table 4 summarizes the used boundary and initial conditions.

**Table 4:** The boundary and initial conditions.

Type	Pressure (Pa)	Temperature (K)	Velocity (m/s)	AOA
Inlet (fixed velocity inlet)	101,325	288	20	from 0° to 28°
Outlet (fixed outlet pressure)	101,325	288	–	–
Initial conditions	101,325	288	20	from 0° to 28°

**Fig. 5:**
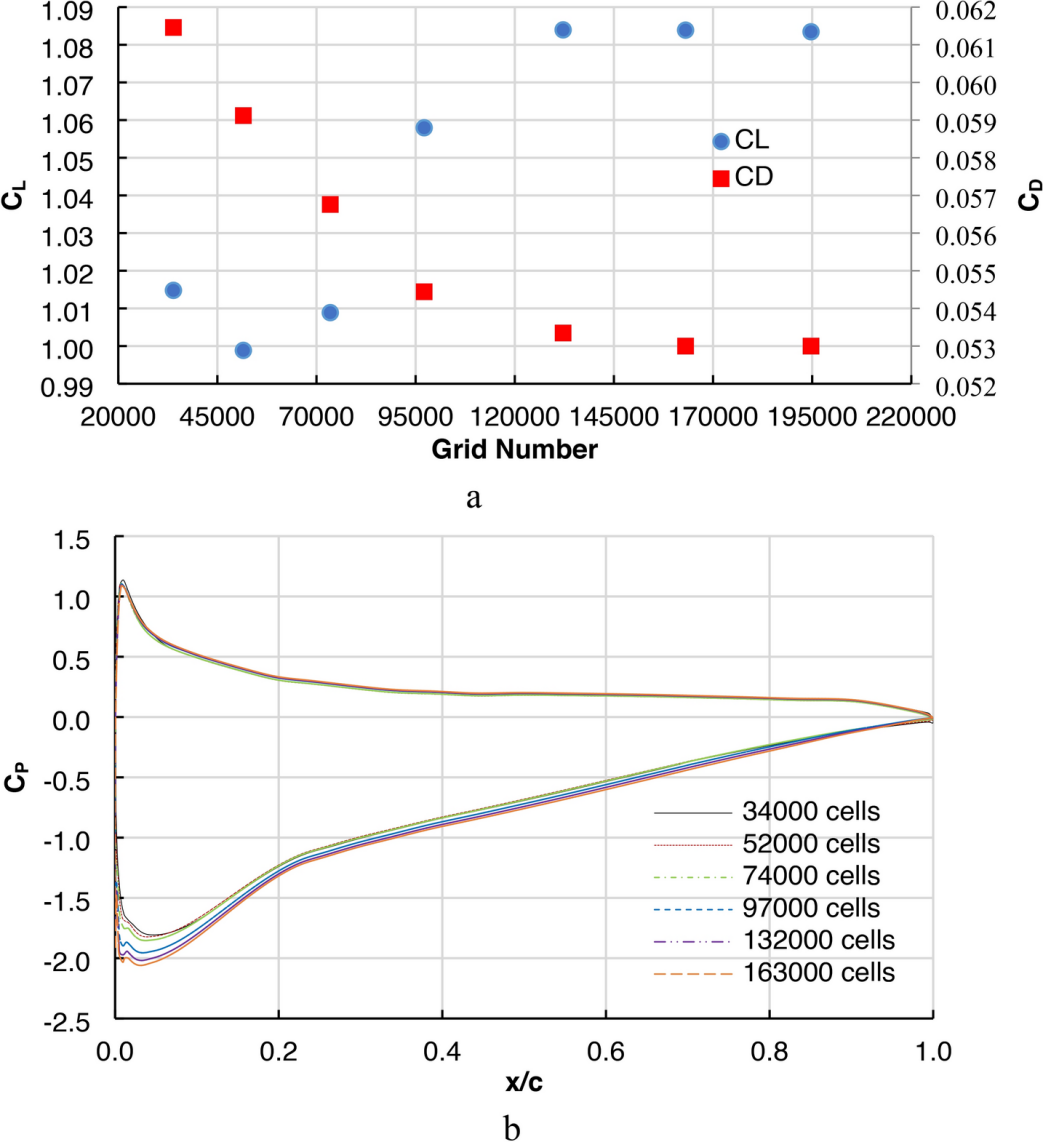
(**a**) Variation of Aerodynamics coefficients with gid numbers, (**b**) Cp variation at AOA 8° with different cell numbers.

A detailed study was conducted to change the grid number and its effect on global calculated parameters such as the lift coefficient and drag coefficient (Fig. 5a). The results showed that the accuracy of the solution was limited to a value less than 0.04% after the number of grids equaled 163,000, so this grid was adopted. The observed flow field variables, such as C_L_ and C_D_, vary with the grid number until the asymptotic numeric value is reached, as shown in Fig. 5a. The effect of the grid number on local computed parameters, such as the distributed values of the pressure coefficient on the airfoil surface, was also studied in Fig. 5b to ensure that the accuracy of the computational grid is sufficiently fine to capture the delicate and important phenomena in the problem. The results demonstrated the high accuracy of the solution with grid number 163000.

### Computational model validation

**Fig. 6:**
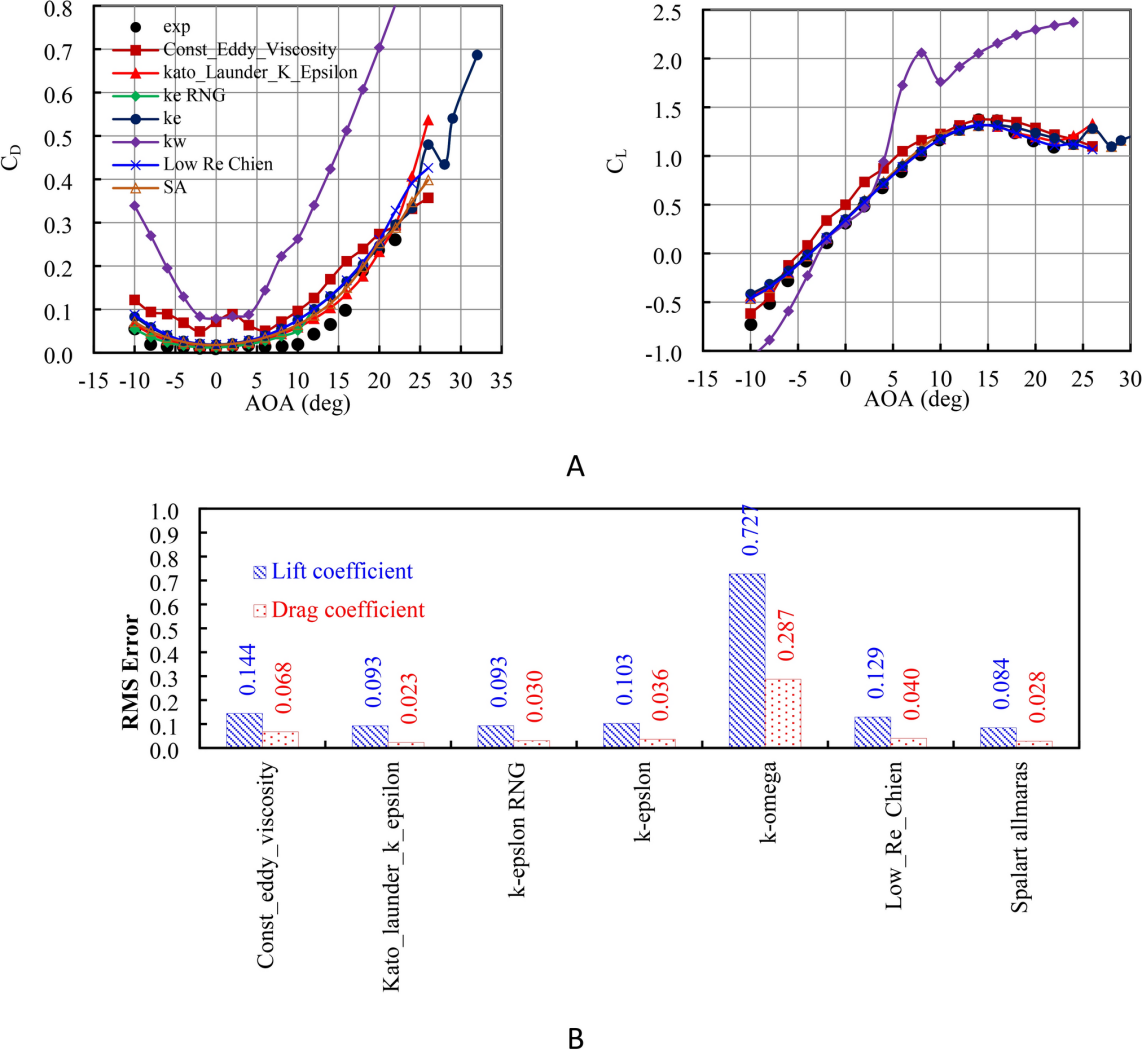
(**A**) The current numerical C_L_ and C_D_ at Re = 1.15 × 10^6^ with several turbulence models compared to experimental measurement by^25^ (**B**) RMS errors for several turbulence models.

This software has been used in several applications in different fields involving aerodynamics^22–24^. Before operating the simulation with the CFD solver, the results of the solver must be validated. Figure 6a presents the validation study results for lift and drag coefficients. The numerical simulation results were compared to the experimental measurements^25^ with different turbulence models at AOA ranging from − 10° to 25° and Re = 1.15 × 10^6^. The results are very similar and indicate high accuracy in calculations, getting close to experimental measurements^25^ in terms of values and trends. Figure 6b illustrates the RMS errors associated with each turbulence model. The error analysis revealed minor discrepancies between the simulation and measurement results, with a root mean square error of 0.084 in C_L_ and 0.028 in C_D_. Nevertheless, the results provided a satisfactory prediction of the overall orientation and stall angle of attack.

In comparing turbulence models to experimental data for stall flow, the Const Eddy Viscosity model captures the stall onset well at AOA = 14° but overpredicts lift and drag post-stall. The Kato–Launder k–ε and k–ε RNG models predict stall accurately at AOA = 14° but slightly underpredict lift post-stall. The k–ε and Low Re Chien models predict an earlier stall and underestimate lift, while the k-omega model predicts an even earlier stall at AOA = 10°, with a rapid lift drop post-stall. The Spalart–Allmaras model matches the stall angle at AOA = 14° but shows a sharp post-stall lift reduction. Overall, predictions vary notably in the post-stall region. Based on the study, the Spalart–Allmaras model was used in the simulations.

## Numerical results and discussion

After completing the study of the accuracy of the numerical solution, a detailed study was conducted of the performance of the slotted airfoil and compared it with the clean airfoil. Figure 6 shows the change in lift and drag coefficients with the change in AOA for the six-slotted airfoil studied cases. The number of slots changes in each case from 1 to 6 respectively. The results are compared to those associated with the clean airfoil to determine the extent of improvement in performance parameters from various perspectives.

**Fig. 7:**
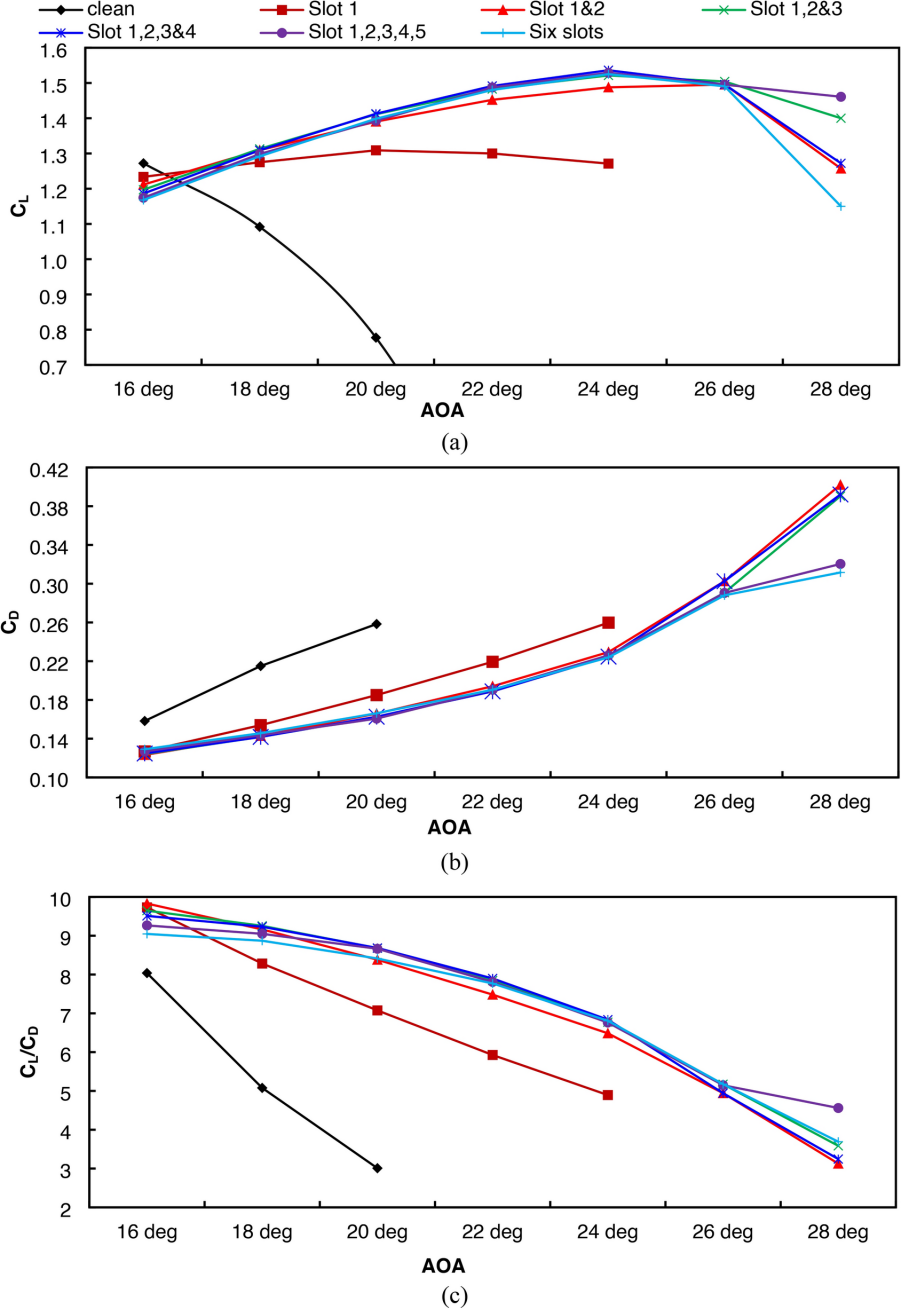
Variation of aerodynamic coefficient with AOA for different slotted airfoil configurations (**a**) C_L_, (**b**) C_D_, (**c**) C_L_/C_D_.

First, for the lift coefficient, see Fig. 7a. In the case of low AOAs, the slot effect is negative, as it was observed that the lift coefficient decreases with the increase in the number of slots. This is because the fluid flows above the clean airfoil and is attached to the upper surface of the airfoil. With the presence of a jet above the surface of the airfoil, an interference occurs between the jet and the incoming air, and this interference results in an undesirable change in the aerodynamic coefficients at a lower AOA. So, the results focus on high AOAs, higher than 16°, as this angle is found to be the stall angle for the clean airfoil. It was observed that, because of the presence of slots, reattachment of the flow occurs because of jetting. It is worth noting here that the zone where the flow separates from the airfoil surface is where the fluid has low energy, so it cannot maintain contact with the surface, which results in a noticeable deterioration in aerodynamic performance, which is undesirable.

The presence of jets from the slots at appropriate speeds because of pumping the fluid from the stagnation point to the slots works to restore the fluid energy and hence the rise in energy level inside the separation zones, which results in the flow reattachment with the surface again. This is of great importance with high AOA and stall angle delays. In most cases, it was found that the presence of the slot helped delay the stall to an AOA equal to 24°, an increase of 10° compared to the clean airfoil, while the airfoil with two slots succeeded in delaying the stall to an AOA equal to 26°, an increase of 12° compared to the clean airfoil. While one slot had the least delay in the stall compared to the other cases, there was an increase of 6° compared to the clean airfoil.

From the point of view of the drag coefficient in Fig. 7b, the slotted airfoil works to reduce the drag coefficient up to an AOA of 20 compared to the clean airfoil. As the AOA increases beyond this, there is a steady increase in the drag coefficient. It was also noted that at AOAs of 26° and 28°, the drag coefficient decreases for the five-slot and six-slot cases compared to the rest of the cases. This discrepancy in results requires displaying the ratio of the lift coefficient to the drag coefficient, which appears in Fig. 7b. The results showed a noticeable improvement compared to the clean airfoil and a decrease in lift values over resistance with increasing AOAs.

The best slot numbers can be inferred from Fig. 7 in terms of cl, cd, or cl/cd at different AOA. From a lift coefficient point of view, the importance of multi-slot passive control started at AOA 18°. The optimum number of slots at this angle is three slots, which increases the lift coefficient by 20.26%. At AOA 20°, the best number of slots is four, with an increase in lift coefficient of 81.6%, followed by three holes with an increase of 81.3%. The jet with four slots is optimal for AOA 22° and 24°. The maximum lift coefficient obtained at AOA 24° with four slot cases is 1.54. However, the three slots succeeded in maximizing the lift at AOA 26°.

From a drag point of view, the passive flow control of different numbers of slots has succeeded in reducing the drag, starting from AOA = 16°. The minimum drag was obtained from two slots with a reduction of 22.1%. At AOA 18°, the four slots succeeded in reducing the drag coefficient by 33.95%. The five slots were superior at AOA 20°, corresponding to a drag reduction of 36.26%. Greater than AOA 22°, the six slots have an advantage over other cases.

Regarding the lift-to-drag ratio of the slotted airfoil, at an AOA of 16°, the comparison between the different cases showed the percentage of cl/cd increment was 22.31% with two slots, while at an AOA of 18°, the percentage increase reached 82.26% with three slots. Starting from AOA 20° up to 24°, the increment reached its maximum value, accompanied by four slots, while the performance of the clean airfoil completely deteriorated. At AOA 24°, the six slots were the best, and on the other hand, five slots were the best at AOA 26°.

There is still a need to explain the differences in results and justifications for changing the values of the aerodynamic performance parameters while changing the number of slots or even just adding them and comparing them to the clean airfoil. Therefore, it was necessary to display the structure of the flow field and the streamlines to clarify the exact locations of separation, the size and intensity of the vortex after separation, and the effect of jetting in several locations on improving performance parameters.

**Fig. 8:**
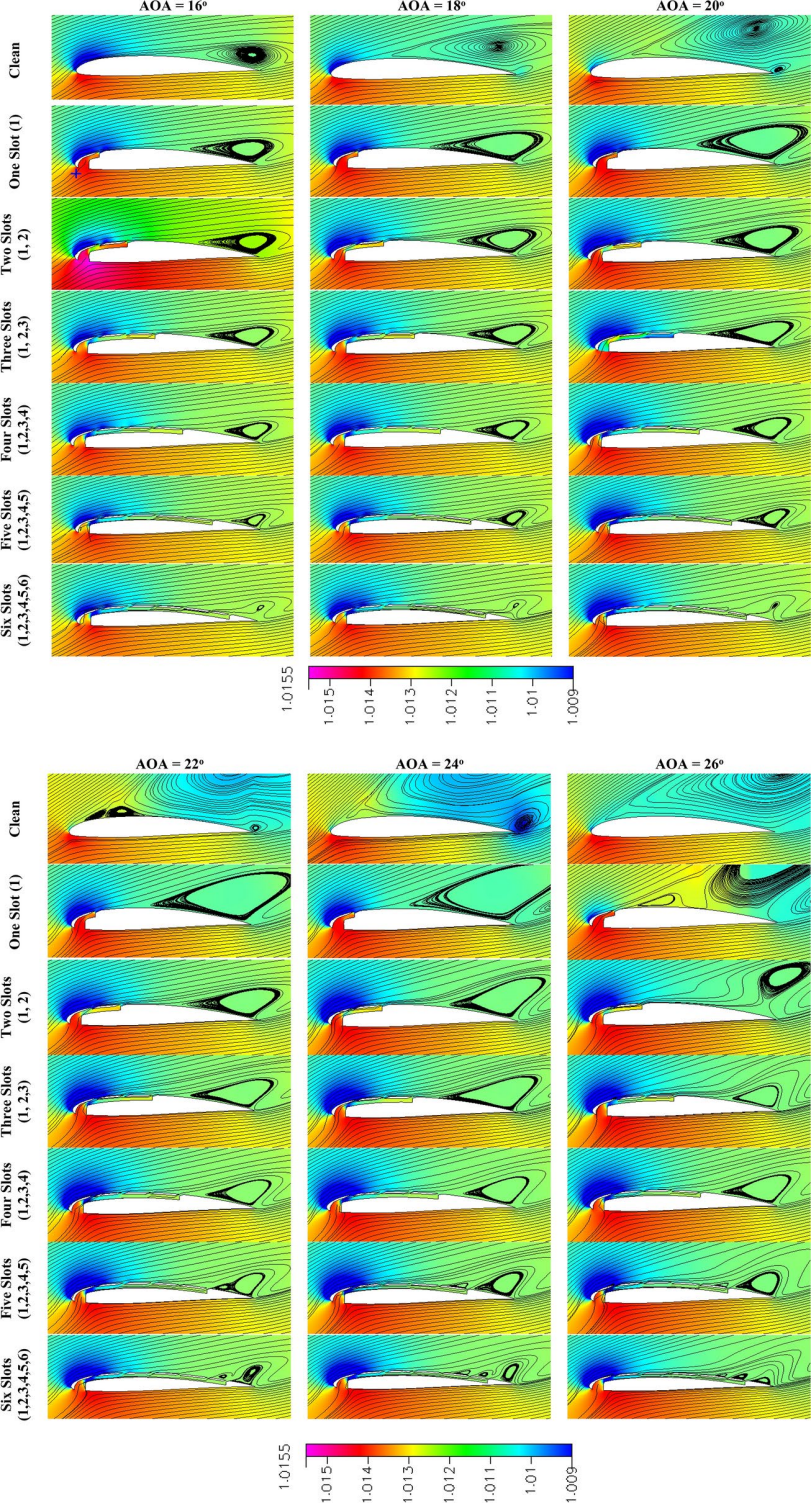
Pressure and streamline contours of clean and slotted NACA 23012C section at different AOAs.

Figure 8 shows a pressure contour and streamlines with changing AOA and changing the number of slots. It is clear from the figure that when the AOA is constant and the number of slots distributed over the surface of the airfoil increases, there is a noticeable effect on the size and intensity of the vortex after separation. Increasing the number of slots reduces and weakens the vortex. By increasing the AOAs, the intensity and size of the vortices increase, but the number of slots has the same effect on the structure of the flow and the streamlines. The number of slots also affects the location of the separation point, as it has been observed that the greater the number of slots, the later the separation point is.

From a drag point of view, separation greatly affects the coefficient of drag generated by pressure, known as form drag. Inside the slot, a change in pressure was observed. The farther it was from the leading edge of the airfoil, the pressure increased, and the velocity decreased, and thus the jet velocity decreased. This affects the flow of energy outside the airfoil’s upper surface, which is added by the jet.

It is worth noting that the amount of energy gained from the slot suction at the entrance is almost constant due to the constant width of the suction in all cases. In addition to its location being fixed in all cases, it is almost at the stagnation point. Thus, the number of slots does not depend on the width of the slot section. The amount of air ingested is distributed over the number of slots, but not equally, as it is affected by the pressure and velocity inside the slot duct. The amount of air injected starts large at the first slot and then decreases successively as it moves away from the leading edge.

**Fig. 9:**
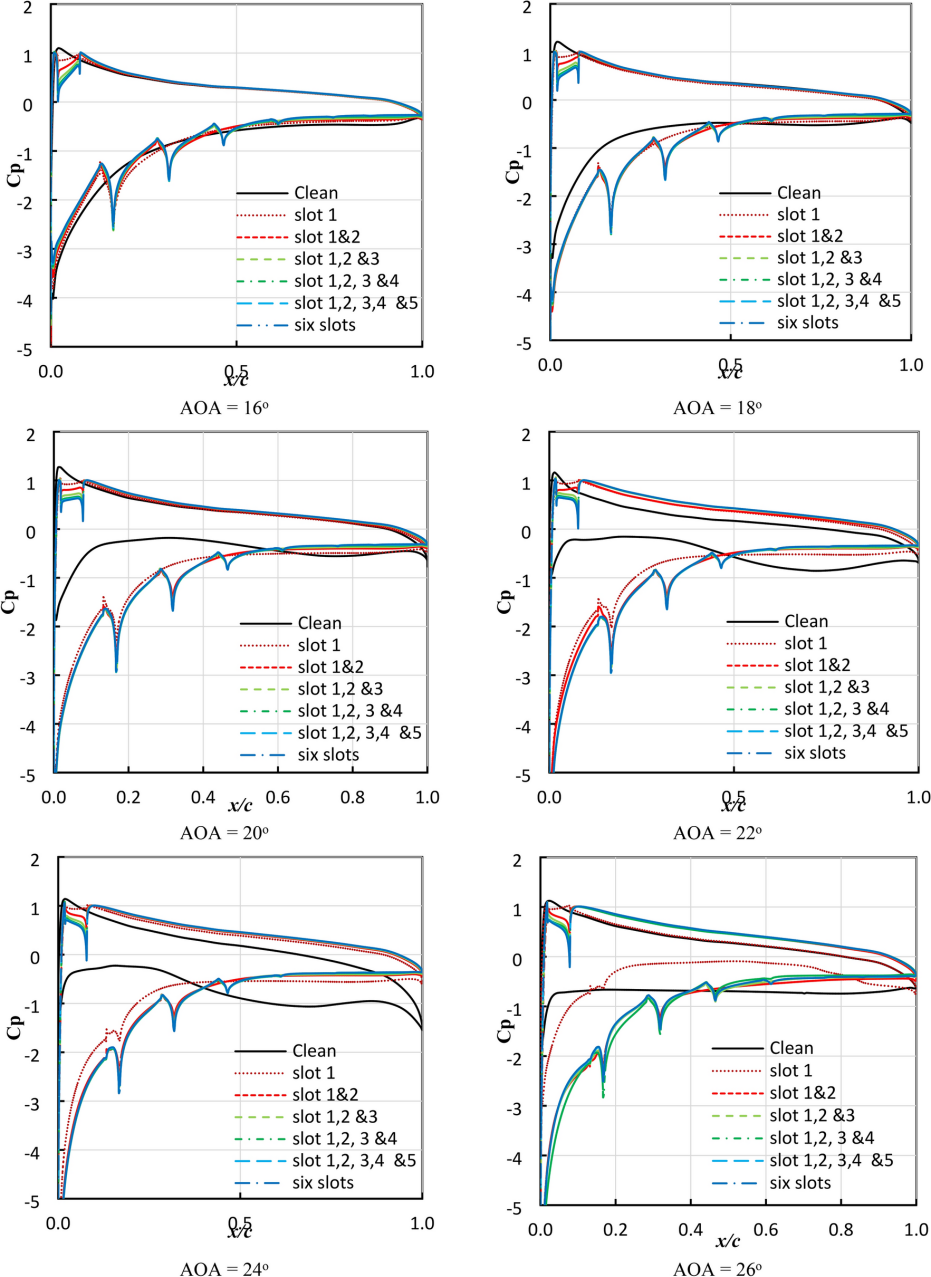
Pressure coefficient distribution of NACA 23012C section at different slot numbers and AOAs.

It is important and necessary to look at the distribution of the pressure coefficient on the airfoil surface, as it is considered the main influence on the values of lift and drag forces. Figure 9 presents the pressure coefficient distribution on both sides of the airfoil. The area enclosed within the pressure coefficient curve is the lift coefficient value. A comparison was made between the pressure coefficient distribution in the case of the clean airfoil and the modified airfoil.

It is clear from the results of the pressure coefficient that at an AOA of 16°, the difference in the enclosed area of the pressure coefficient curve is almost non-existent, as there is an identity between the two curves with the presence of the effect of jetting at the first, second, and third slots. This effect shows a decrease in the value of the pressure coefficient on the suction side, and this decrease occurs suddenly at the point of jetting then, the pressure coefficient values return to increase again completely instantaneously after the point of jetting.

The decrease in the pressure coefficient in the presence of the slot on the suction side appears clearly in the locations of the jet (the slot exit) in cases with one, two, and three slots. On the other hand, in cases with four, five, and six slots, the pressure coefficient decreases at jet locations 4, 5, and 6. This confirms what was previously concluded: the flow rate is at its maximum value at the first jet and decreases successively in the following jet locations.

What can be concluded from the results obtained is that there is an increase in the enclosed area of the pressure coefficient curve, and this increment is proportional to increasing AOA up to the stall angle. It is worth noting here that there is an advantage after an AOA of 18° if the slotted airfoil is compared to the clean airfoil. The stall AOA changes in different numbers of slots, and this was mentioned earlier.

The results were analyzed from the point of view of the distribution of the pressure coefficient on the suction and pressure sides of the airfoil in terms of the number of slots and the change in AOAs. Confirmation of what was previously analyzed was achieved. The deduction of the stall AOA, which is achieved by any slot number, is presented.

In the case of the single slot, there is a clear increase in the enclosed area up to an AOA of 20°. At an angle of 20°, starting from the flow does not provide energy close to the surface of the airfoil to help flow attachment to the surface. This confirms what was concluded previously and the occurrence of the stall at an angle of 20°. After that angle, starting from AOA 22°, the values of the pressure coefficient decrease for both sides of the airfoil from x/c = 0.4–1.0.

With regards to the two slots, there is a clear increase in the enclosed area up to an AOA of 26°. At an angle of 26°, there is an increased enclosed area within the pressure coefficient curve compared to the airfoil with a single slot. This confirms what was concluded previously.

For the other cases, it was found that the improvement (increase in an enclosed area) compared to the clean airfoil extends up to an AOA of 24° for the different cases except for the single and double slots. Before x/c of 0.4, for AOA less than 24°, the difference in coefficient of pressure between pressure and suction sides increases compared to a clean airfoil. It is also clear that there is no improvement from the point of view of the pressure coefficient above three slots.

**Fig. 10:**
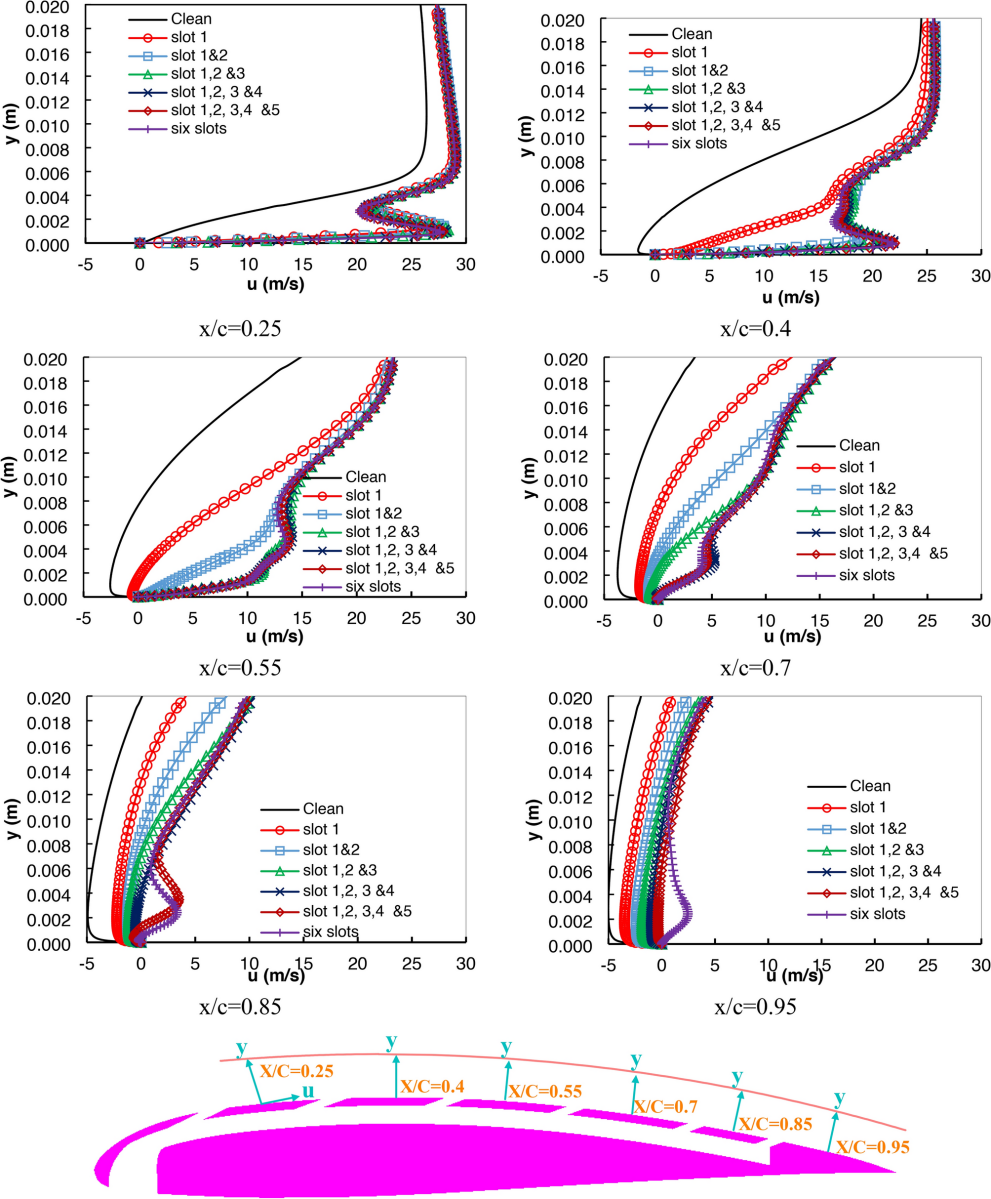
Velocity profile at six locations along the airfoil suction side with AOA 18°.

**Fig. 11:**
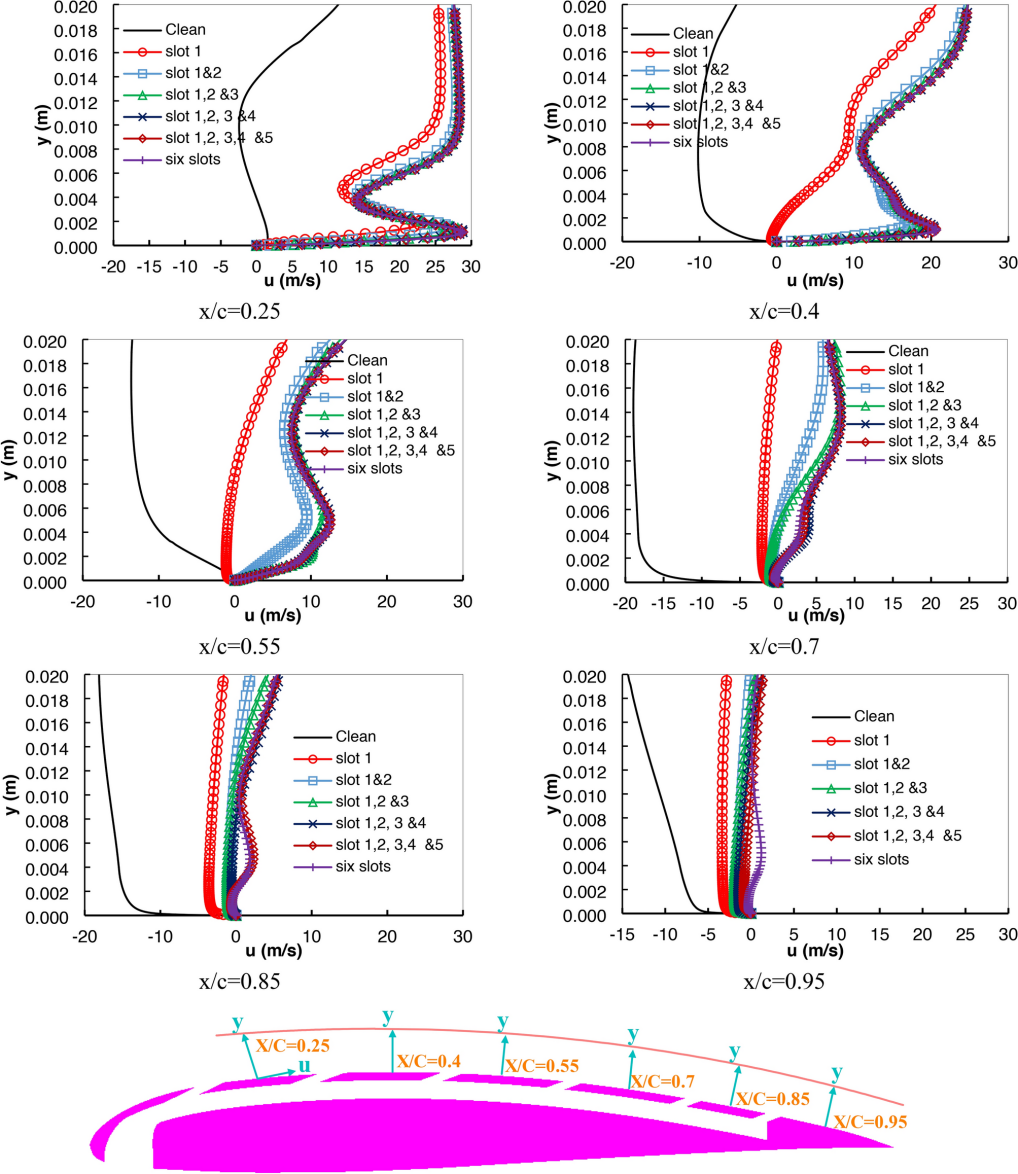
Velocity profile at six locations along the airfoil suction side with AOA 24°.

Figures 10 and 11 display velocity profile curves at several locations along the airfoil suction side, specifically after each slot location. This section presents a deep analysis of the phenomenon that arises from the mixing and interaction of main flow and synthetic jet flow, which did not appear in previous analyses. The locations were specified at x/c = 0.25, 0.4, 0.55, 0.7, 0.85, and 0.95. The curves were drawn at high AOAs, 18° and 24° specifically. The angles were chosen after the stall occurred in the clean airfoil so that the difference became clear because of adding the slot.

Figure 10 shows the velocity profile curves at an AOA of 18°. The maximum velocity magnitude increased by existing the slot at different locations. At a location x/c = 0.25, it was found that the velocity curves are all positive, which shows that there is no possibility of separation occurring in the clean and different slot cases. While at the location x/c = 0.4, that is, just after the second slot location, a clear separation occurred in the case of the clean airfoil only. The presence of the slots succeeded in preventing separation from occurring starting from this location, although the single slot showed a different profile from the other cases. This is explained by the existence of a profile location far from the jetting location, where the jetting effect was weakened, resulting in a weakening of the flow energy close to the surface at this location.

At x/c = 0.55, separation and reversal flow velocities were observed for the clean and one slotted airfoil with a lower intensity. The two-slot airfoil succeeded in eliminating the existence of reverse flow and separation despite the weak flow energy near the surface due to the distance of the velocity profile location from the second jet location. The other cases maintained the same energy level at the same velocity profile location without separation because the slot opening was located directly before the profile location.

At x/c 0.7, the four, five, and six slotted airfoils succeed in eliminating the reverse flow at that location compared to the clean and other slotted airfoils. Regarding x/c = 0.85 for the five and six-slotted airfoils, there is a small value for negative velocities very close to the airfoil surface. Although moving away from the airfoil surface, the velocities become positive, and the flow is reenergized due to the existence of jets before that location. The six slotted airfoils were the only ones that maintained a high energy level of flow along the airfoil surface up to x/c = 0.95.

Figure 11 describes the velocity profile curves at a higher AOA of 24°. At a location x/c = 0.25, it was found that the velocity curves are all positive except for the clean airfoil. This shows that there is no possibility of separation occurring in the different slot cases. While at the location x/c = 0.4, that is, just after the second slot location, a clear separation occurred in the case of the clean and one-slotted airfoil. The other cases of the slots succeeded in preventing separation from occurring starting from this location. This is explained by the existence of a profile location far from the jetting location, where the jetting effect was weakened.

The velocity profiles at x/c = 0.55 are close to location x/c = 0.25, except the separation and reverse flow velocities were increased for the one-slotted case. The velocity profiles at x/c = 0.7, 0.85, and 0.95 revealed that the slotted airfoils succeeded in decreasing the high reverse flow at those locations compared to the clean airfoil.

## Comparison of proposed slotted airfoil configurations

Table 3 compares the multi-slotted airfoil proposed configurations to improve the airfoil’s passive aerodynamic performance when compared to the clean one. The flow separation and stall phenomena begin early for the clean airfoil, beginning with an AOA of 14°, while the slots work to inject air into the weak areas, delaying the separation angle until 20° in the case of one slot, 26° in the case of two slots, and 24° in the rest of the studied slots. Delaying separation by slotting the airfoil improves the lift coefficient while decreasing the drag coefficient, resulting in better lift-to-drag ratios. Because the clean airfoil completely stalls after AOA of 20°, and thus its lift value is zero after AOA of 20°, the aerodynamic performance of slotted and clean airfoils was compared at AOA of 20°, as shown in Table 5. The lift coefficient increases from 68.4% in the case of one slot to 81.8 percent in the case of four slots, while the drag coefficient decreases from 28.5% in the case of one slot to 37.8% in case of five slots, increasing the lift to drag ration by a rate ranging from 135.9% in case of one slot to 189.5% in case of four slots.

**Table 5:** Summary of results for clean and slotted airfoils with different slot numbers.

Airfoil configurations	Area (m^2^) 10^−4^	Area reduction %	Cost saving %	Lift enhancement % at AOA 20°	Drag reduction % at AOA 20°	Cl/Cd enhancement % at AOA 20°	Stall AOA (°)
Clean	34	–	–	–	–	–	14
Single slot (1)	31.7	6.75	3.2	68.4	28.5	135.9	20
Two slot (1,2)	30.22	11.11	5.2	78.9	35.9	179.5	26
Three slot (1,2,3)	28.74	15.46	7.3	81.6	37.1	189.3	24
Four slot (1,2,3,4)	27.26	19.82	9.3	81.8	37.1	189.5	24
Five slot (1,2,3,4,5)	25.78	24.17	11.4	79.2	37.8	188.9	24
Six slot (1,2,3,4,5,6)	24.79	27.08	12.7	80	35.7	180.5	24

Not only does using multi-slotted airfoil improve the dynamic performance of wind turbine blades without consuming energy, but it also reduces the cross-sectional area of the airfoil and thus the amount of materials used in manufacturing the blade, which is reflected in the weight of the wind turbine rotors and its economic cost. According to what the National Renewable Energy Laboratory (NREL) previously approved, the cost of raw materials used in the manufacture of wind turbine blades accounts for approximately 47% of the total cost of the turbine blade [26 & 27]. As a result, at AOA of 20°, the current multi-slotted airfoil provides an advantage in improving lift coefficient, reducing drag coefficient, and thus improving wind turbine performance with a lift-to-drag ratio of up to 180.5%. The amount of blade materials is reduced by approximately 27.08%, lowering the total cost of the turbine blades by approximately 12.7%. Using a four-slot airfoil reduces blade material by approximately 19.8%while saving the total cost of turbine blades by approximately 9.3%.

## Conclusion

The present study focused on multi-slotted passive flow control to cover the overall suction side surface with energized flow from the pressure side, specifically near the stagnation point. The definition of the new synthetic multi-slotted airfoil proposed in the current study represents the novelty of the current work. In this study, the aerodynamic performance of a multi-slot NACA23012C airfoil was investigated as a passive flow control approach to enhance wind turbine blade efficiency. The analysis was conducted numerically at a Reynolds number of 2.74 × 10^5^, focusing on the effects of varying the number of airfoil slots from one to six. The methodology included a numerical grid optimization study, validation with experimental results from the literature, and a detailed flow field analysis to assess the aerodynamic benefits. The following points present the most important and prominent comments from the findings of the current study.

- A multi-slot airfoil ensures that air is injected along the suction side of the airfoil, allowing stalls to be delayed by 6°–12° relative to the clean airfoil.
- The velocity profiles revealed that the slotted airfoils succeeded in eliminating or decreasing the high reverse flow at different locations along the airfoil compared to the clean airfoil.
- When compared to a clean airfoil, the maximum lift coefficient increment was 15.8% with the four-slot case. The multi-slotted airfoil reduced drag over a wide range of AOA, beginning at 16°. The maximum C_L_/C_D_ increment from two slots across the AOA range of 16°–26° was 22.31%.
- The presence of jets from the slots at appropriate speeds helps to restore energy levels within the separation zones. The slot jet flow rate is greatest at the first jet and gradually decreases at subsequent jet locations.
- Increasing the number of slots has a noticeable effect on the size and intensity of the vortex after separation, but only up to 4 slots; increasing more than four slots does not affect lift.
- Starting with AOAs higher than 22°, it is preferable to use several slots rather than four slots to obtain a lower drag coefficient.
- At low AOAs, the slot effect is negative, as it was observed that the lift coefficient decreases with the increase in the number of slots.
- Using the optimum number of slots (4 slots), a multi-slotted airfoil reduces the cost of the blade material by about 19.8%, lowering the total cost of the turbine blades by about 9.3%.

## Data Availability

The data presented in this study is available on request from the corresponding author.

## List of symbols

C_L_: Lift coefficient
C_D_: Drag coefficient
C_P_: Pressure coefficient
C_L_/C_D_: Lift-to-drag ratio
c: Airfoil chord (m)
_e_: Specific internal energy (per unit mass) (J/kg)
F: Body force (N)
_k_: Thermal conductivity (W/m^2^ °C)
$\dot{m}$: Mass flow rate (kg/s)
_P_: Pressure (N/m^2^)
_Q_: Internal heat generation (J/m^3^)
_qr_: Radiation heat flux vector (W/m^2^)
T: Temperature (K)
U: Velocity (m/s)
$$\vec{V}$$: Velocity vector (m/s)
ρ: Air density (kg/m^3^)
_μ_: Viscosity (Pa s)
_Φ_: Mechanical or viscous dissipation function (W/m^3^)
